# Exploring the inhibitory effect of membrane tension on cell polarization

**DOI:** 10.1371/journal.pcbi.1005354

**Published:** 2017-01-30

**Authors:** Weikang Wang, Kuan Tao, Jing Wang, Gen Yang, Qi Ouyang, Yugang Wang, Lei Zhang, Feng Liu

**Affiliations:** 1 State Key Laboratory of Nuclear Physics and Technology, School of Physics, Peking University, Beijing, People’s Republic of China; 2 Center for Quantitative Biology, Peking University, Beijing, People’s Republic of China; 3 Beijing International Center for Mathematical Research, Peking University, Beijing, People’s Republic of China; Tel Aviv University, ISRAEL

## Abstract

Cell polarization toward an attractant is influenced by both physical and chemical factors. Most existing mathematical models are based on reaction-diffusion systems and only focus on the chemical process occurring during cell polarization. However, membrane tension has been shown to act as a long-range inhibitor of cell polarization. Here, we present a cell polarization model incorporating the interplay between Rac GTPase, filamentous actin (F-actin), and cell membrane tension. We further test the predictions of this model by performing single cell measurements of the spontaneous polarization of cancer stem cells (CSCs) and non-stem cancer cells (NSCCs), as the former have lower cell membrane tension. Based on both our model and the experimental results, cell polarization is more sensitive to stimuli under low membrane tension, and high membrane tension improves the robustness and stability of cell polarization such that polarization persists under random perturbations. Furthermore, our simulations are the first to recapitulate the experimental results described by Houk *et al*., revealing that aspiration (elevation of tension) and release (reduction of tension) result in a decrease in and recovery of the activity of Rac-GTP, respectively, and that the relaxation of tension induces new polarity of the cell body when a cell with the pseudopod-neck-body morphology is severed.

## Author summary

The coupling of mechanical and chemical factors has been consistently shown to regulate a variety of cellular processes such as cell polarization, migration and fate determination. However, most of the modeling work on biological systems still focuses on chemical systems. Here, we established a minimal cell polarity model coupling the two factors by linking the interaction between F-actin polymerization and tension with the action of the Rac signaling pathway in cell polarity. We performed single cell measurements to test the predictions of this model regarding the spontaneous cell polarization of two types of cancer cells with different cell membrane tensions. Membrane tension modulated the spatiotemporal properties of polarity and adjusted the cells’ sensitivity to external stimuli. Furthermore, our model recapitulates the experimental results described by Houk *et al*., indicating that an increase (decrease) in tension results in a decrease (recovery) of Rac-GTP activity and that the relaxation of tension induces new polarity of the cell body when a cell with the pseudopod-neck-body morphology is severed. The framework of our model may be naturally extended to explore mechanical effects on other complex biological networks.

## Introduction

Cell polarity, a cell state with an asymmetric distribution of specific molecules and organelles along a geometric axis (‘front to back”) in cell morphology [1–4], is essential for various kinds of cell functions, including migration [3] and asymmetric division [4]. For example, cell polarization mediates cell migration and the protrusions that extend in direction of migration [3]. Cell polarity involves both chemical systems and mechanical systems [4]. The chemical systems include the cytoskeleton, surface receptors, and polarity proteins [5, 6]. Among the chemical systems, GTPases (Rho proteins) play key roles in cell polarization during cell movement [7]. Activated Rac (Rac-GTP) mainly concentrates at the leading edge of the polarized cell [3]. Moreover, it induces actin polymerization and protrusions at the leading edge [7, 8]. The mechanical factors consist of the force and stress properties of the extracellular matrix (ECM), cytoskeleton and membrane [4]. For instance, ECM geometry determines the direction of cell polarity [9]. Stochastic actin shell rupture induces the formation of one leading edge due to the relaxation of tension [4]. The global actin cytoskeleton interacts with the membrane and modulates membrane tension [10, 11].

Furthermore, the chemical system is often coupled with mechanical factors during cell polarization. For example, mechanical stress down-regulates lamellipodia formation by inhibiting Rac [12, 13]. Houk and coworkers showed how membrane tension regulated cell polarity in HL-60 cells [14]. As shown in their aspiration-release experiment, Rac-GTP activity decreased upon aspiration and subsequently recovered after release. In the severing experiment, HL-60 cells formed a tethered morphology with a pseudopod, a long and thin neck and a cell body following brief heat shock. The long, thin neck severely restricted the diffusion-based communication between the pseudopod and the cell body. Surprisingly, the cell body grew new protrusions in tens of seconds if the neck was cut. This result contradicted the common assumption that cell polarity was generated by inhibitors diffusing from the polarized front, suggesting that membrane tension might be the long-range inhibitor [14].

A series of mathematical models of cell polarization have been proposed as a special case of pattern formation in biological systems [15]. Heterogeneous spatial patterns were suggested to arise from simple reaction-diffusion systems in the pioneering paper by Turing [16]. Meinhardt applied a generic reaction-diffusion model to form polar structures induced by local activation balanced with global inhibition [17]. Another method using a local excitation, global inhibition (LEGI) model was proposed by Levchenko and Iglesias [18]. The common assumption of these models is that cell polarity is generated by the interaction between two types of molecules: self-activated, slow diffusing molecules and global inhibiting, fast diffusing molecules. Notably, the wave pinning (WP) model provides a minimal reaction-diffusion system with bi-stable kinetics to pin the waves into a stable polar distribution [19, 20]. This model is based on the exchange between the active, membrane-bound form and inactive, cytosolic form of an important polarity protein, Rac [3]. These models, however, neglected the effects of mechanical factors. Moreover, the models addressing mechanical systems of polarity mainly studied the interaction between membrane tension and F-actin [21, 22] but lacked the interaction between membrane tension and the upstream network in chemical reaction systems. To the best of our knowledge, very few studies have attempted to integrate both chemical reactions and mechanical systems. Previous models are insufficient to explain the aspiration-release experiment and severing experiment [14].

Here, we present a mechano-chemical model of cell polarity by incorporating membrane tension with reaction-diffusion systems. Based on the WP model, we combine the feedback between Rac and F-actin with the repression of actin polymerization by membrane tension [4, 23–25]. The model generates stable polarity once stimuli exceed certain thresholds. Moreover, polarity is reversed or steered by new stimuli. According to the simulations, membrane tension affects the polarization time and the sensitivity to the attractant, and higher membrane tension leads to better robustness and stability of cell polarization in response to random perturbations. Consistent with the model, the single cell experiment conducted using CSCs and NSCCs indicates that the former has lower membrane tension and subsequently tends to undergo spontaneous cell polarization and change directions. Moreover, for the first time, our mechano-chemical model explains the results of the aspiration-release and severing experiments [14].

## Results

### The mechano-chemical model of cell polarization

**Fig 1:**
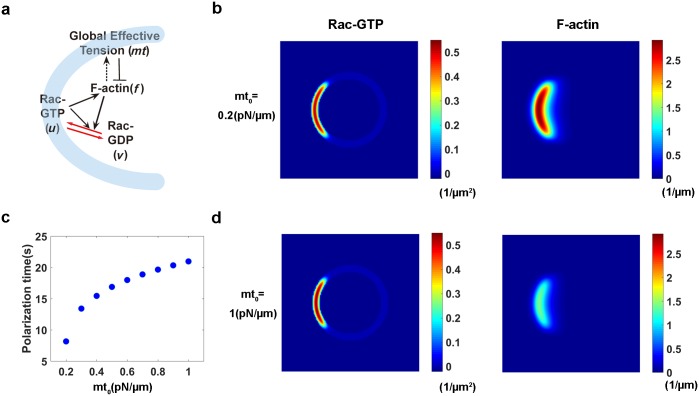
The mechano-chemical model of cell polarization. **(a)** Schematic diagram of the regulatory network in the model. Membrane tension (*mt*) globally inhibits (denoted by—**|**) the formation of F-actin (*f*) in the cell. F-actin polymerization increases (black dashed arrow) membrane tension, and activates (black solid arrow) membrane-bound Rac-GTP (colored in light blue) (*u*). Rac-GTP is also activated by stimulation and itself. In addition, Rac-GTP on the membrane and Rac-GDP (*v*) in the cytosol interconvert (red arrow). **(b)** The steady-state spatial profiles of Rac-GTP, Rac-GDP and F-actin in a polarized cell with *mt*_*0*_ = 0.2 *pN/μm*. Rac-GTP and F-actin congregate at the front of the cell in the membrane and cytoplasm, respectively. **(c)** Increased membrane tension elongates the polarization time in response to the same stimulus. **(d)** As *mt*_*0*_ increases to 1 *pN/μm*, the distribution of Rac-GTP shrinks, and the maximum concentration of Rac-GTP increases.

We establish a minimal cell polarity model incorporating the interactions between Rac-GTP, Rac-GDP, F-actin and membrane tension (Fig 1a). The conversion between Rac-GTP and Rac-GDP is formulated by adopting the WP model (Equations 1 and 2). The feedback loop between F-actin and Rac-GTP is complicated and regulated by various enzymes [24, 26]. The negative feedback from F-actin to Rho GTPase (such as Rac-GTP) was incorporated in the WP model; a variety of patterns from static polarization to actin wave formation are observed as the feedback strength increases [27]. Moreover, positive feedback from F-actin to Rac-GTP has also been proposed to exert a gradient-amplifying effect. This hypothesis has been confirmed in multiple experiments [24, 26, 28–32]. A Hill function (Equations 1 and 2) is applied in the model to explore the effect of this positive feedback on cell polarization. In addition, we assume that effective membrane tension (*mt*) functions as a global negative regulator to attenuate F-actin polymerization since the equilibrium of the force on the membrane is on the time scale of milliseconds, which is much faster than the diffusion-based chemical reaction [11]. For simplification, we also assume that *mt* is a function of the total amount of F-actin (Equation 3) as membrane tension is usually primarily determined by the membrane-associated cytoskeleton (actin cortex) instead of the plasma membrane itself [33]. Thus, F-actin effectively has a negative feedback effect on itself and on Rac-GTP activation.

Instead of treating the cell as a projection of the membrane and cytoplasm on one line or plane, similar to the traditional one- or two-dimensional (2D) cell polarity models [19, 20, 34, 35], here, we propose a phase field model, which has been widely used to model vesicle bio-membranes [36] and cell motility [37–39]. By introducing the phase field function to distinguish the interior of the cell from the exterior, the membrane position is naturally determined by the diffuse layer of the phase field function (Equation 4). This function allows the model to account for the different positions of Rac-GTP on the cell membrane and Rac-GDP and F-actin in the cytosol (Fig 1a and Equations 5, 6 and 7). We also use an alternative approach that incorporates a traditional 2D cell polarity model coupled with membrane tension to test the robustness of the mechano-chemical mechanism. We assume this 2D cell presents the projection of a 3D cell on one plane; hence, the cell membrane overlaps with cell cytosol. The regulation of F-actin by membrane tension is described by applying the Brownian ratchet model [25] (S1 Equation) rather than Hill functions (see S1 Text).

We first confirm that both models are able to capture the common features of cell polarization shown in previous models [19]. First, cells are observed to spontaneously polarize in response to noise, i.e., a random distribution of stimuli (S1a and S1b Fig) and in response to gradients (Fig 1b and 1d and S1c and S1d Fig). Rac-GTP and F-actin mainly concentrate at one end of the cell after cell polarization (Fig 1b and 1d, and S1 Fig), whereas Rac-GDP is nearly evenly distributed across the cell with a concentration of *v*_0_ due to its greater diffusion rate. The maximum and minimum concentration of Rac-GTP are the two stable solutions *u*_*H*_ and *u*_*L*_ for (∂*u* / ∂*t* = 0) when *v* equals *v*_0_. Second, in the experiments, cells sustain their polarity without external stimuli. Indeed, the asymmetric distribution of Rac-GTP is maintained after the stimulation is removed in our simulations. Third, consistent with the experimental results showing that a polarized cell could redirect its movement in a different direction in response to a new stimulus [19], the polarized cell could shift its polarity by 90 degrees (S1 Movie) or reverse its polarity when it is treated with a transient stimulus presented from another direction. Finally, in response to two simultaneous stimuli with different amplitudes, two polarization fronts initially form in the cell, and subsequently, the front triggered by the stronger stimulus finally ‘absorbs’ the other front (S2 Movie).

More importantly, according to our mechano-chemical model, membrane tension strongly influences the spatiotemporal characteristics of the cell polarity. Regarding the temporal characteristics, the polarization time (which is defined as the duration from the initiation of the stimulus to the time when the concentration of Rac-GTP reaches a higher steady value *u*_*H*_) increases as membrane tension increases in response to the same stimulus (Fig 1c). Regarding the spatial characteristics, a lower density of Rac-GTP disperses in a larger area in cells with lower membrane tension (Fig 1b and 1d), i.e., polarized cells with higher membrane tension have sharper fronts.

### A lower membrane tension increases the tendency of the cell to polarize

The Rac-GTP concentration may never reach *u*_*H*_ if membrane tension is above a certain value in response to the same stimulus, suggesting the existence of a threshold of the amplitude (*ks*_*amp*_) and duration (*ks*_*dur*_) of the stimulus. Hence, we simulated the Rac-GTP and F-actin dynamics at a specific membrane tension by fixing the duration and varying the amplitude (S2a Fig). Indeed, the cell is only able to exhibit stable polarity as the maximum concentration of Rac-GTP increases towards the higher stable value *u*_*H*_ if the amplitude is sufficient. However, when the amplitude is below a certain value, the maximum concentration of Rac-GTP gradually decreases to the lower stable value *u*_*L*_ after transiently increasing to a value below *u*_*H*_, indicating that the cell does not polarize under this condition. The F-actin results are similar to the Rac-GTP results. The simulation results are similar to the content discussed above when we vary the durations while fixing the amplitudes (S2b Fig).

**Fig 2:**
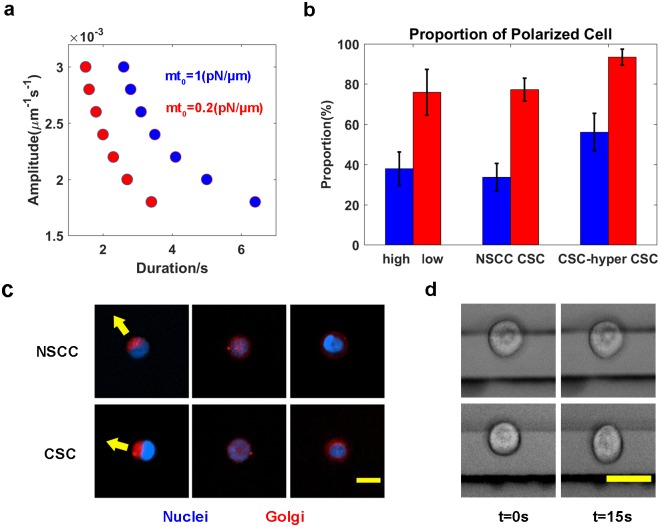
A lower membrane tension increases the tendency of the cell to polarize. **(a)** Threshold relationship between the amplitude and duration of stimuli for inducing cell polarity at different values of membrane tension. **(b)** The comparison of the proportion of polarized cells with low and high membrane tension in the simulation (left panel), of CSCs and NSCCs (middle panel), and in the experiment examining CSCs cultured in hypotonic medium (right panel). Error bars represent the standard deviation. **(c)** Representative images of polarized (left column) and nonpolarized (right two columns) NSCCs (top) and CSCs (bottom) on circular ECM patterns. Scale bar: 20 *μm*. In polarized NSCCs and CSCs, the Golgi (red) aggregates on one side of the nucleus (blue). However, in the nonpolarized NSCCs and CSCs, the Golgi (red) disperses throughout the cell (middle) or forms a ring around the nucleus (blue). **(d)** Comparison of relative cell deformation of NSCCs (top) and CSCs (bottom). Scale bar: 20 *μm*. Both CSCs and NSCCs have a round shape, as they are trapped by the electrodes (the two horizontal bars) with an electric field of 2 *V* (left). CSCs elongate much more than NSCCs along the direction of the electric field after the electric field is increased to 5 *V* for 15 *s* (right).

We further calculated the thresholds for the amplitudes and durations for cells with different membrane tensions. Under the same duration (amplitude) of stimulus, the amplitude (duration) threshold increases as the membrane tension increases (S2c and S2d Fig). We also calculated the stimulus threshold by varying both the amplitude (*ks*_*amp*_) and duration (*ks*_*dur*_) for *mt*_*0*_ from 0.2 to 1 (Fig 2a). The *ks*_*dur*_ *vs*. *ks*_*amp*_ curve shifts away from the origin as membrane tension increases. Thus, cells with lower membrane tension respond to weaker stimuli polarize, consistent with our hypothesis that membrane tension serves as a global inhibitor of cell polarization. As predicted, cells with lower membrane tension have a higher tendency to polarize (Fig 2b, left) in response to the same random stimuli (Equation 9).

We tested the prediction of this model by measuring the differences in cell polarization in CSCs and NSCCs (Fig 2b, middle). The Golgi was aggregated in CSCs and NSCCs sorted from MCF-7 cells (Fig 2c), which are known to show dispersed Golgi [40], and we confirmed that the polarized distribution of Golgi was highly correlated with the cell migration direction (S3 Fig). Furthermore, the initiation of cell polarization triggers the restricted localization of the Golgi at the front side of the polarized cell, and, in turn, secretion from the Golgi toward the proximal plasma membrane domain helps to maintain cell polarity [41]. In addition, the morphology and position of the Golgi are importantly related to the accumulation of F-actin (cell protrusion) in migrating cells [42]. Hence, for the MCF-7 cells in our experiment, the morphology of the Golgi served as a surrogate for the usual cell polarity markers, such as the distribution of Rac or F-actin. Of the cells grown on circular ECM patterns without any inducer gradients (S3 Fig), the proportion of polarized CSCs is 77.3±5.7% (mean±standard deviation from 3 measurements, the number of cells in each experiment is *N* = 53, 75 and 81), more than a two-fold increase compared with the polarized NSCCs (33.7±6.9%, *N* = 57, 83 and 95) (Fig 2b, middle). Thus, CSCs undergo spontaneous polarization more easily than NSCCs.

Based on the prediction of our mechano-chemical model, CSCs would have a lower membrane tension than NSCCs. Therefore, we examined the membrane tension of CSCs and NSCCs using a cell deformation device. As the cell shape reaches the stable state under an applied electric field, NSCCs show little change in shape, but the CSCs exhibit significant deformation (Fig 2d). The relative elongation ($\frac{a_{t=15s}-a_{t=0}}{a_{t=0}}\times 100\%$, *a* denotes the cell length along the direction perpendicular to the edges of the electrodes) of CSCs is 14.9±5.6% (*N* = 10), which is much greater than the value for the NSCCs (3.7±3.1%, *N* = 13). Moreover, the elongation index ($EI=\frac{a-b}{a+b}\times 100\%$, where *b* represents the cell length along the direction parallel to the edges of the electrodes) is 13.3±2.4% for CSCs compared to 5.9±2.8% for NSCCs. Based on these results, Young’s modulus of CSCs is less than the value for the NSCCs [43]. Hence, the membrane tension of CSCs is less than the NSCCs, as membrane tension is proportional to Young’s modulus [44].

We further performed a test experiment to determine whether the proportion of the polarized CSCs will decrease if cell membrane tension is reduced. The addition of myosin inhibitors [12], stretching the elastic substrate to which the cells attach, or aspirating cell membrane with a micropipette [12, 14] can modify membrane tension. We chose to alter membrane tension by varying the osmotic pressure [13], a convenient way to modulate a population of cells. As the CSCs were immersed in hypotonic medium (DMEM/F12 diluted with the same volume of double distilled water), their membrane tension is expected to increase; hereafter, these cells are called CSC-hyper [13]. The proportion of polarized CSCs decreases from 93.4±4.0% (*N* = 80, 71 and 40) in iso-osmotic DMEM/F12 medium to 56.2±9.3% in hyperosmotic medium (*N* = 66, 68 and 46) (Fig 2b, right). These experimental results are consistent with the prediction of the model that higher membrane tension decreases the sensitivity of cells’ polarity in response to stimuli. Moreover, the proportion of polarized CSCs in DMEM/F12 is greater than the proportion in DMEM with 10% FBS (Fig 2b, middle); hence, factors other than membrane tension also affect the polarization. More experiments, such as manipulating F-actin polymerization with drugs, are required to validate these findings and exclude the effects of other factors.

### Membrane tension stabilizes polarization in response to perturbations

**Fig 3:**
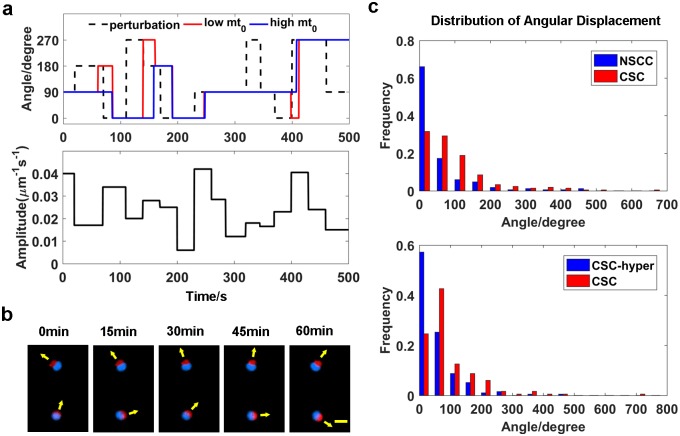
Membrane tension affects the dynamics of cell polarity. **(a)** When the stimulus switches between four directions, i.e., 0, 90, 180, 270, (top, black dashed line) and varies in its amplitude (bottom), higher initial membrane tension (top, red) improves the stability of the polarized cell compared to lower initial membrane tension (top, blue). **(b)** Representative images of the cell polarity dynamics. Scale bar: 20 *μm*. **(c)** Histogram of the angular displacement of polarized CSCs and NSCCs (top), as well as CSCs and CSC-hyper (bottom).

Because higher membrane tension requires a greater stimulus to polarize a cell, we expect that high membrane tension, in turn, will stabilize polarization in response to perturbations. We tested this hypothesis by determining how the cell responds to random stimuli. Stimuli with random amplitudes and durations in random direction are added to the simulations after the cell polarizes. Then, we compare the number of the times the stimulus redirects (*n*_*s*_) and the number of the times the polarity redirects (*n*_*p*_). If the initial membrane tension is higher, polarity is redirected less frequently (average *n*_*p*_ / *n*_*s*_ = 0.59±0.07) comparing with the cells with a lower initial membrane tension (average *n*_*p*_ / *n*_*s*_ = 0.80±0.10) (Fig 3a), suggesting that high membrane tension improves the stability of the polarized cell.

We also tested the predictions of this model with single cell measurements and confirmed that the polarity of CSCs varies more easily than the polarity of NSCCs. Polarized cells could change the orientation of their polarity on circular ECM patterns (Fig 3b). We measured the distributions of the angular displacements of CSCs and NSCCs in one hour (Fig 3c, top). We define the rotation angle in 5 minutes as the angular speed. The angular displacement is the total angular speed observed in one hour. According to the distribution of angular displacements, the direction of polarized CSCs varies to a greater extent than the polarized NSCCs and CSC-hyper (Fig 3c, bottom). In fact, most polarity vectors of NSCCs only vibrate around the initial positions. Hence, the polarity of CSCs changes more easily than NSCCs, i.e., CSCs are more sensitive to perturbations. If the perturbation is from the external environment, CSCs could shift their polarity toward the direction of the perturbation or the source of attractant in a more timely manner, which is critical for metastasis.

### Simulations of the aspiration-release and severing experiments

**Fig 4:**
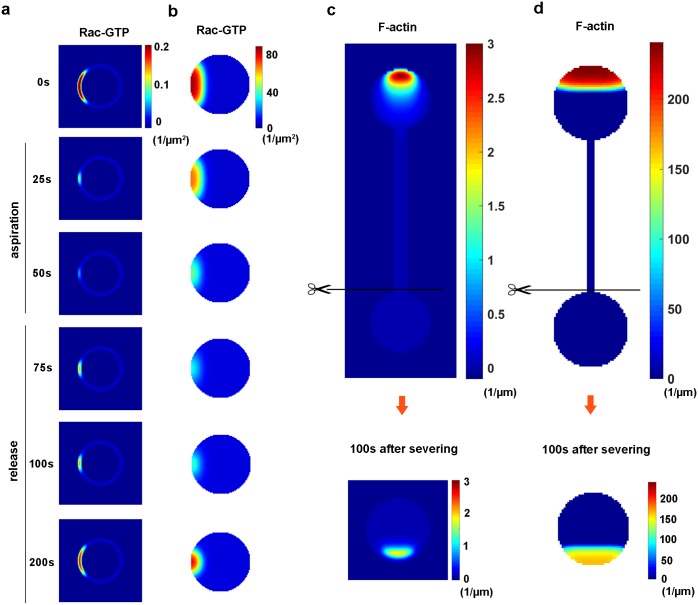
Simulations of the aspiration-release experiment and the severing experiment using the cell polarity model with phase field formulation (a and c) and the traditional cell polarity model coupled with membrane tension (b and d). **(a-b)** Rac-GTP dynamics in the polarized cell decrease during aspiration (elevated membrane tension) due to the inhibition of Rac-GTP. After release (a sudden decrease of membrane tension), the cell recovers its active Rac-GTP levels and regains its polarity. **(c-d)** A polarized cell forms a steady morphology with a pseudopod, neck and body. F-actin only concentrates at the front of the pseudopod. When the pseudopod and the neck are severed, the cell exhibits new polarity in the nonpolarized rear cell body.

We further conducted simulations to explain the experimental results reported by Houk and coworkers, which have not yet been mimicked by the conventional reaction-diffusion systems. First, according to the aspiration-release experiment, the activity and concentration of Rac-GTP are reduced upon aspiration and sequentially recovered upon release [14]. To numerically implement this experiment, we assume that the process of aspiration and release occurs instantly, as the details of the tension changes are unclear. As shown in the simulation, if membrane tension is increased upon aspiration, the concentration of Rac-GTP in polarized cells decreases in approximately 50 *s* (Fig 4a). Subsequently, the relationship between membrane tension and F-actin is resumed when aspiration is released. Approximately 150 *s* are required for the cell to recover the activity of Rac-GTP. The traditional cell polarity model coupled with membrane tension shows similar results, albeit a slight delay for the cell to recover the activity of Rac-GTP (Fig 4b). Thus, our model closely captures the characteristics of the cell that loses and regains its polarity in the aspiration-release experiment.

Our model also successfully solves another puzzle, the pseudopod-neck-body morphology severing experiment. In the simulation, cell occurs in the tethered morphology (Fig 4c, top). F-actin is evenly distributed in the rear cell body at a very low concentration. Since the nonpolarized rear cell body cannot exhibit new polarity without an uneven distribution or perturbation from the internal or external environment, we maintained the random stimuli (production of Rac-GTP, Equation 9) below a certain threshold throughout the simulation. Although this perturbation does not destabilize the polarized cell with a tethered morphology, it does induce polarity, i.e., asymmetric distribution of F-actin (Fig 4c, bottom), in the nonpolarized rear cell body upon severing when membrane tension of the cell body is reduced to preserve the relationship with the F-actin concentration. In contrast, the cell body does not reanimate if membrane tension is maintained at the same levels before and after severing (S4 Fig). These results were also reproduced with the traditional cell polarity model coupled with membrane tension (Fig 4d). Thus, for the first time, our model recapitulates the phenomena observed in the pseudopod-neck-body morphology severing experiment.

## Discussion

### Coupling of mechanical factors and the chemical system

The key of the proposed model for cell polarity is the successful coupling of the chemical systems and membrane tension based on the molecular interactions. In particular, we choose F-actin to link membrane tension and Rac, the key molecule regulating cell polarity. On one hand, we incorporate F-actin polymerization into the Rho GTPase dynamics, based on the positive feedback loops between F-actin and Rac-GTP (Fig 1a, [4, 24]). On the other hand, we couple membrane tension with the F-actin polymerization dynamics [25]. Although more detailed gene regulation and protein interaction mechanisms are neglected [24], this minimal model successfully captures the general features of cell polarity, similar to the other models [19]. For example, our model utilizes the stable polarized state, the threshold of stimuli for polarization, and responses to new stimuli. In addition, the stimulus with greater strength increases membrane tension to a greater extent in our new model; consequently, the final stable polarity state has a sharper front (Fig 1d). However, in the WP model, the final polarized state is the same once the stimulus exceeds a certain threshold [19]. Furthermore, the proposed model overcomes the limitations of the previous cell polarity models, which are only based on reaction-diffusion systems [18, 20, 45] or mechanical systems [21, 22]. This model allows us to explore the effects of membrane tension on cell polarity in simulations. For example, membrane tension affects not only the spatial profile of cell polarity but also the sensitivity of the cell to external stimuli. One of the most important achievements of this proposed model is that it recapitulates the aspiration-release experiment and the pseudopod-neck-cell body morphology severing experiment for the first time [14].

Physical forces, such as effective membrane tension and the components regulating cell polarity, form a web of reciprocal interactions [4]. In addition to the interactions formulated in our current model, other possible interactions could also be explored [1, 4]. Membrane tension has been suggested to directly feed back onto the kinetics of polarity-regulating proteins, e.g., membrane tension and curvature are sensed by Bar domain proteins and feed back onto the Rac-GTP activation/deactivation rates [33].

For simplification, the current model neglects the effects of cell shape and the curvature of the membrane on cell polarity [46–48]. Moreover, we assume that membrane tension is homogeneous, although there may be inhomogeneous membrane tension [33, 49]. Furthermore, the dependence of membrane tension on the F-actin concentration may be more complicated than the simple linear relationship formulated in Equation 7. All these factors described above will be naturally incorporated into our cell polarity model with phase field formulation in future studies to further improve our model. In addition, if we consider that increasing contacts between cells and the extracellular matrix could increase the stiffness and viscosity of the actin cytoskeleton [50], the model could be extended to studies of the dynamics of cell polarity by coupling chemotaxis and mechanotaxis.

### Free energy landscape of cell polarity

From the point of view of the free energy landscape, cell polarization is regarded as an inter-conversion between the nonpolarized state and the polarized state. Since membrane tension affects the threshold of the polarization stimulus (Fig 2), we naturally postulate that membrane tension modulates the height of the potential barrier between the two states (S5 Fig). The stimulus acts as a driving force by elevating the nonpolarized state to a high-energy state. Only sufficient stimulation above a threshold will help the cell conquer the potential barrier and achieve the polarized state. Although we have not defined the accurate height of the potential barrier because the proposed reaction-diffusion model is not a gradient system [51], one may still be able to construct the quasi-potential and qualitatively estimate the barrier height by calculating the strength of the stimulus, i.e., the product of the amplitude and the duration of the stimulation. The curve of the threshold duration for different amplitudes of stimulus under stable membrane tension (Fig 2a) exhibits a hyperbola-like profile. The product increases 1.8-fold as *mt*_*0*_ increases from 0.2 to 1 *pN/μm*.

### Sensitivity and persistence of cell polarity

Based on our model, cells utilize an extra parameter, membrane tension, in addition to the biochemical reaction parameters to tune the sensitivity and persistence of cell polarity. Cells must achieve a delicate balance between the sensitivity of detecting the external gradient and the persistence of the directed migration during chemotaxis to adapt to the complex environment. Cells with a low membrane tension have the advantage of detecting the low external gradient of an attractant. Subsequently, the increase in membrane tension during polarization could prevent the cell from reacting to other perturbations with the same strength (S5 Fig). One advantage would be to avoid the formation of multiple polarization fronts, which often leads to a time-wasting elimination of the extra fronts [20]. The other advantage would be to maintain persistent cell movement in one direction until new polarity forms in response to a stronger stimulus [52]. Both features indicate a better search strategy for cells.

Interestingly, this search strategy might have been exploited by CSCs, which are believed to be responsible for tumor metastasis [53]. In addition to resistance to radiotherapy and chemotherapy [54], CSCs exhibit greater migration and deformation [55] than NSCCs. Since deformation is related to the cytoskeleton, among which F-actin is the most resistant protein to deformation [56], and the actin cytoskeleton is the main determinant of membrane tension [33], CSCs may have a lower membrane tension. Indeed, we confirmed that CSCs deform more easily than NSCCs in the single cell deformation measurements. Based on our model, CSCs exhibit higher polarization sensitivity and a better searching strategy during migration. This prediction was further confirmed in the single cell experiment showing that the proportion of spontaneously polarized CSCs is increased more than two-fold compared with the NSCC. These results may facilitate the development of a new therapeutic strategy for tumor metastasis by targeting the signaling pathways regulating the membrane tension of tumor cells.

Mechanical factors have been increasingly appreciated for the significant roles they play in diverse cellular processes by interacting with chemical systems. For example, advection and stress affect the spatial patterns in morphogenesis [57]. Tension activates ion channels on the membrane [33]. The stiffness of the substrate plays significant roles in differentiation [58]. To fully understand these biological systems, researchers must surpass the reaction-diffusion models and establish coupling models that integrate both chemical reaction and mechanical systems. Our proposed model in this study provides a minimal system that incorporates membrane tension into biochemical polarization systems. This framework could also be naturally extended to explore the other complex biological networks.

## Materials and methods

### Cell polarity model with phase field formulation

The cell is postulated to exhibit a round shape Ω_0_ with a radius *R* = 10 *μm*, which is of the actual size and is contained in a larger computational domain Ω with the size of 40 *μm* *40 *μm*.

We extend the WP model to describe the inter-conversion between active Rac-GTP on the membrane ∂Ω_0_ and inactive Rac-GDP in the cytosol Ω_0_, as well as their interactions with F-actin polymerization in the cytosol Ω_0_ (Equations 1, 2 and 3). The concentrations of Rac-GTP, Rac-GDP and F-actin are denoted as *u*, *v* and *f*, respectively. *D*_*u*_, *D*_*v*_ and *D*_*f*_ are diffusion coefficients with *D*_*u*_ < *D*_*f*_ ≪ *D*_*v*_. The basal conversion rate from Rac-GDP to Rac-GTP is *b* and the rate at which Rac-GTP is dephosphorylated to Rac-GDP is *r*. The positive self-feedback of Rac-GTP is described by a Hill function $\frac{cu^{2}}{u^{2}+K^{2}}$. We add a Hill function term to Equations 1, 2 and 3 to account for the positive feedback loop between Rac-GTP activation and F-actin polymerization. In addition, *c*_*i*_ and *K*_*i*_ (i = 1,2,3) represent the maximum self-activation rate and the microscopic dissociation constant, respectively. Likewise, the rate of F-actin depolymerization is denoted as *d*_*f*_. The formula for the negative self-feedback effects of membrane tension on F-actin is based on two assumptions: 1) membrane tension is a function of the total amount of F-actin ($mt\left(f\right)=mt_{0}(1+\lambda \int_{\Omega_{0}}fds)$, where *mt*_*0*_ is the basal value of membrane tension in the nonpolarized state, *λ* represents the dependence of *mt* on the amount of F-actin, and 2) the rate of F-actin polymerization is down-regulated by membrane tension, which is inversely proportional to $1+\frac{mt(f)}{K_{F}}$, where *K*_*F*_ is a scaling factor for nondimensionalization.

Based on the WP model and assumptions listed above, the dynamics of the model system are described using the following equations:

$$\{\begin{array}{lll} \frac{\partial u}{\partial t} & =D_{u}\nabla^{2}u+\left(b+\frac{c_{1}u^{2}}{u^{2}+K_{1}^{2}}+\frac{c_{2}f^{2}}{f^{2}+K_{2}^{2}}\right)v-ru, & \left(1\right)\\ \frac{\partial v}{\partial t} & =D_{v}\nabla^{2}v-\left(b+\frac{c_{1}u^{2}}{u^{2}+K_{1}^{2}}+\frac{c_{2}f^{2}}{f^{2}+K_{2}^{2}}\right)v+ru, & \left(2\right)\\ \frac{\partial f}{\partial t} & =D_{f}\nabla^{2}f+\left(\frac{c_{3}u^{2}}{u^{2}+K_{3}^{2}}\cdot \frac{K_{F}}{K_{F}+mt(f)}\right)-d_{f}f, & \left(3\right) \end{array}$$

We apply a phase field function to label the intracellular and extracellular regions and to distinguish Rac-GTP (*u*) on the cell membrane ∂Ω_0_ from Rac-GTP (*v*) and F-actin (*f*) in the cytosol Ω_0_.

$$\begin{array}{ccc} \varphi \left(x,y\right) & =\frac{1}{2}\left(\text{tanh}(\frac{R-\sqrt{x^{2}+y^{2}}}{\varepsilon })+1\right),\;\;\;\;\left(x,y\right)\in \Omega & \left(\text{4}\right) \end{array}$$

Hence, the cell membrane is described by a narrow transition layer between the interior of cell (*φ*(*x*,*y*) = 1) and the exterior of cell (*φ*(*x*,*y*) = 0) with a width of *ε*. The position of the cell membrane is approximated using the function *B*(*φ*) = *φ*^2^(1 − *φ*)^2^ or |∇*φ*|, which vanishes outside the narrow interface. Thus, the phase field function is naturally used to account for Rac-GTP on the cell membrane and Rac-GTP and F-actin in the cytosol in a regular computational domain Ω.

By coupling the phase field function with the WP model, Equations 1, 2 and 3 are replaced with the following equations:

$$\{\begin{array}{llll} \frac{\partial B(\varphi )u}{\partial t} & =D_{u}\nabla \cdot \left(B\left(\varphi \right)\nabla u\right)+B\left(\varphi \right)\left(b+\frac{c_{1}u^{2}}{u^{2}+K_{1}^{2}}+\frac{c_{2}f^{2}}{f^{2}+K_{2}^{2}}\right)v-B\left(\varphi \right)ru, & in\;\;\Omega & \left(5\right)\\ \frac{\partial \varphi v}{\partial t} & =D_{v}\nabla \cdot \left(\varphi \nabla v\right)-\left|\nabla \varphi \right|\left(b+\frac{c_{1}u^{2}}{u^{2}+K_{1}^{2}}+\frac{c_{2}f^{2}}{f^{2}+K_{2}^{2}}\right)v+\left|\nabla \varphi \right|ru, & in\;\;\Omega & \left(6\right)\\ \frac{\partial \varphi f}{\partial t} & =D_{f}\nabla \cdot \left(\varphi \nabla f\right)+\left|\nabla \varphi \right|\left(\frac{c_{3}u^{2}}{u^{2}+K_{3}^{2}}\cdot \frac{K_{F}}{K_{F}+mt(f)}\right)-\varphi d_{f}f, & in\;\;\Omega & \left(7\right) \end{array}$$

Here, the terms in the cytosol are coupled with *φ* and the terms on cell membrane are coupled with *B*(*φ*) or ∇*φ*. Phase field coupling has been proven to be a consistent approach for modeling the transport, diffusion and adsorption/desorption of material quantities on a deformable interface [59], and has been successfully applied to many biological models, such as vesicle bio-membranes [36] and cell motility [37–39].

The external stimulus is added in Equations 5 and 6 to increase the rate of transformation from Rac-GDP to Rac-GTP. In simulations, the stimulus is expressed as *k*_*s*_*v*, where *k*_*s*_ is spatially dependent. We fix the spatial relation of the external stimulus and only vary its amplitude (*ks*_*amp*_) and the duration (*ks*_*dur*_) of *k*_*s*_ to simplify the assumption. The following expressions of the stimulus are used:

The global graded stimulus *k*_*s*_ is

$$\begin{array}{ccc} k_{s}^{grad} & =\{\begin{array}{l} ks_{amp}(R\pm x)\;or\;ks_{amp}(R\pm y),\;0\leq x\leq R,\;0\leq y\leq R,\;0<t\leq ks_{dur},\\ 0,\;t>ks_{dur}, \end{array} & \left(\text{8}\right) \end{array}$$

The local random stimulus *k*_*s*_ is

$$\begin{array}{ccc} k_{s}{}^{loc} & =\{\begin{array}{l} \{\begin{array}{ll} ks_{amp}\cdot R_{0}, & 0\leq x\leq 0.25R\;or\;0\leq y\leq 0.25R\\ 0, & else, \end{array}0<t\leq ks_{dur},\\ 0,\;t>ks_{dur}, \end{array} & \left(\text{9}\right) \end{array}$$

where *R*_0_ is a uniformly distributed random number between 0 and 1.

The values and units for all parameters are listed in S1 Table. The diffusion coefficients *D*_*u*_, *D*_*v*_ and *D*_*f*_ are set according to published values [60, 61]. The maximum self-activation rate of Rac-GTP *c*_1_ and dephosphorylation rate *r* are chosen based on published values [20, 62]. We use parameter fitting to determine the values of the other parameters, including Hill coefficients and microscopic dissociation constants (*K*_*i*_, *i* = 1, 2 or 3), as well as the rate of F-actin degradation (*d*_*f*_) and maximum activation rates for Rac-GTP and F-actin (*c*_*2*_ and *c*_*3*_). We also perform sensitivity analyses for all parameters to investigate the effects of the values of these parameters on cell polarity (S6 Fig).

The initial conditions affect the spatial profile of Rac-GTP. If the initial concentrations of Rac-GTP and Rac-GDP are too low, their distributions are unlikely to break symmetry and therefore the cell remains nonpolarized, regardless of the strength of the stimulus. Initial homogeneous concentrations of *u* = 2 *μm*^−2^, *v* = 6 *μm*^−2^, *f* = 0 are used to represent the initial nonpolarized state for the simulation of cell polarization. We also analyze the influence of the initial conditions in our simulations (S7 Fig). If we use a stochastic distribution as the initial Rac-GTP and Rac-GDP by adding a Gaussian distribution noise with a mean of 0 and a variation of 0.01, the steady-state spatial profiles of Rac-GTP and Rac-GDP are consistent (the maximum concentration of Rac-GTP is 0.5210 ± 1.4853 × 10^−5^ *μm*^−2^ and the total amount of Rac-GTP is 92.5253 ± 0.0934).

We choose the periodic boundary condition and apply the efficient semi-implicit Fourier-spectral method for spatial discretization to solve Equations 5, 6 and 7 [63].

We also developed another traditional cell polarity model coupled with membrane tension by assuming that the cell membrane overlaps with cell cytosol in 2D to further investigate the effect of membrane tension on cell polarity. In this model, we applied the Brownian ratchet model [25] to describe the mechanism by which membrane tension regulates F-actin. The simulations yielded consistent results with the phase field formulation, supporting the negative effect of membrane tension on cell polarization (S1c and S1d Fig, Fig 4b and 4d). Please see more details in the Supporting Information. Values for the parameters used in this model are shown in S2 Table.

### Cell line and cell culture

The MCF-7 breast cancer cell line was purchased from American Type Culture Collection (ATCC). MCF-7 cells were cultured in Dulbecco’s Modified Eagle’s Medium (DMEM) supplemented with 10% fetal bovine serum, 100 *μg/mL* penicillin, and 100 *μg/mL* streptomycin at 37°C in a 5% CO_2_ atmosphere mixed with 95% air. Cells were stained with anti-CD44-PE (BD Pharmingen), and anti-CD24-Alexa Fluor 488 (BioLegend) antibodies for sorting with a flow cytometer (BD FACS Aria II). The CD44^+^/CD24^-^ phenotype was regarded as CSCs, whose proportion was approximately 1.6% [64]; the other three phenotypes, CD44^+^/CD24^+^, CD44^-^/CD24^+^, and CD44^-^/CD24^-^, represented the NSCCs [65, 66]. CSCs sorted from MCF-7 cells were cultured in DMEM/F12 (1:1 mixture of DMEM and Ham’s F-12 medium) with 20 *ng/mL* basic fibroblast growth factor (bFGF), 10 *ng/mL* epidermal growth factor (EGF), B27 serum-free supplement and N-2 supplement to inhibit the differentiation of CSCs [64]. The culture dish was incubated with Geltrex (120–180 *μg/mL* at 37°C for an hour before use. The phenotype of CSCs was maintained for at least one month.

### Cell deformation

Two planar microelectrodes composed of indium tin oxide on glass slides with a 20 *μm* gap were ablated with ultraviolet (UV) light. A PDMS trough was mounted onto the processed slide, which could accommodate approximately 200 *μL* samples. Cells were detached from the bottom of dishes using trypsin/EDTA (Sigma-Aldrich) and suspended in a mixture of a 0.3 M inositol solution and phosphate-buffered saline (PBS). The electric conductivity of the solution was adjusted to 2 *mS/m*. Two hundred microliters of cell suspension were added to the device. A square waveform signal with an amplitude of 2 *V* and a frequency of 12 *MHz* generated by a function generator (AFG3021B, Tektronix) was applied to the electrodes to drive the cells’ translational motion and trap them between the gap of the electrodes due to the dielectrophoresis process [67]. The trapped cells were stretched by adjusting the amplitude of the square wave to 5 *V* for 15 *s* and then restored by decreasing the voltage to 2 *V* over the next 15 *s*. Time-lapse images were captured with a microscope (IX81, Olympus) using a 40X objective with a numeric aperture (NA) of 0.6 every 33 *ms* for 30 *s*.

### Fabrication of micro-patterns

Surface micro-patterns were fabricated using the degassing-assisted patterning method [68]. The diameter of the circular pattern was 25 *μm* and the interval was 90 *μm*. The non-fouling material used for micro-patterning was poly L-lysine-graft-poly ethylene glycol (PLL-g-PEG) [69]. PLL-g-PEG attaches to negatively charged surfaces, such as the surface of a glass slide treated with oxygen-plasma, and the surface will be resistant to the adsorption of proteins and attachment of cells [70]. A glass slide (Fisher Brand) was treated with oxygen-plasma for 3 minutes. The glass slide was covered with a piece of polydimethylsiloxane (PDMS) with arrays of pillars (diameter = 25 *μm*) and degassed for 5 minutes. Then, the PDMS mold and the glass slide were returned to atmospheric pressure and PLL-g-PEG (0.1 *mg/mL* in 10 *mM* HEPES) was added to the edge of the PDMS mold. PLL-g-PEG was aspirated into the space between the PDMS mold and the glass slide. After a 1 hour incubation (attachment of PLL-g-PEG on the glass slide), the PDMS mold was removed from the glass slide. The glass slide was air-dried [71]. A piece of PDMS (12 *mm*×5 *mm*) covered the slide when the slide was treated with plasma to avoid plasma-induced damage to the micro-patterns. The profile of the PDMS was depicted on the other side of the slide to mark its position. The glass slide with a bulk PDMS with a small well (length: 12 *mm*, width: 5 *mm*, and height: 5 *mm*) was treated with plasma and bonded together by fitting the well with the marked profile (S1a Fig). Two hundred microliters of a mixture of fibronectin (an ECM protein, 25 *μg/mL*) and fibrinogen-Alexa Fluor 488 (*10 μg/mL*, Invitrogen) was added to the small well, incubated for 1 hour and rinse with phosphate-buffered saline (PBS) three times.

### Imaging and image processing

Before loading, cells cultured in the 24-well plate were stained with 1 *μg/mL* Hoechst 33342 for 20 minutes at 37°C and rinsed with PBS. Subsequently, they were stained with 333 *μg/mL* Golgi-Tracker Red (Molecular Probes) at 4°C for 30 minutes, rinsed with ice-cold medium and incubated in fresh medium for 30 minutes at 37°C. Then, the cells were loaded into the small well (S1a Fig) for spontaneous polarization measurements and into confocal culture dishes to compare the orientation of cell polarity and the direction of migration. Time-lapse imaging was performed on a Zeiss inverted microscope (Axio Observer.Z1 (SP)) every 5 minutes for at least 1 hour. The objective was 20X with an NA = 0.5 in air. The detection channels included bright field, DAPI (excitation at 352 *nm* and emission at 455 *nm*) and mCherry (excitation at 587 *nm* and emission at 610 *nm*) using an X-cite lamp.

### Hypotonic medium for increasing membrane tension

Before being loaded into the small well, CSCs were suspended in a hypotonic medium (a 1:1 mixture of DMEM/F12 medium and double distilled water), which increased their membrane tension [13]. Images were captured after four hours when the cells adhered to the micro-patterns.

## Supporting Information

S1 Text Traditional cell polarity model coupled with membrane tension. (PDF)

S1 Fig Steady-state spatial distribution of Rac-GTP and F-actin. **(a-b)** Steady-state spatial distribution of Rac-GTP **(a)** and F-actin **(b)** during the spontaneous polarization of a cell in response to noise simulated using the cell polarity model with phase field formulation. **(c-d)** Steady-state spatial profiles of Rac-GTP **(c)** and F-actin **(d)** in a polarized cell in response to a transient gradient stimulus calculated using the traditional polarity model.
(TIF)

S2 Fig Membrane tension affects the threshold of the stimulation required for cell polarization. **(a)** The dynamics of the maximum Rac-GTP concentration at a fixed duration and varying amplitudes of the stimuli. When the amplitude of the stimulus is below a threshold, the cell cannot polarize (blue line). The other lines show the polarization dynamics as the amplitudes exceed the threshold. **(b)** The dynamics of the maximum Rac-GTP concentration in the stimulation with varied durations and a fixed amplitude. When the duration of the stimulus is below a threshold, the cell cannot polarize (black line). The other lines show the polarization dynamics as the durations exceed the threshold. **(c)** For duration-fixed stimuli, the threshold of the stimulation amplitude required for polarization increases as membrane tension increases. **(d)** For amplitude-fixed stimuli, the threshold of the stimulation duration increases as membrane tension increases.
(TIF)

S3 Fig Single cell polarization measurements. **(a)** Pictures of the microstructure chip and images of the fluorescent, coated ECM patterns. Scale bar: 20 *μm*. **(b)** The orientations of polarized CSCs and NSCCs are uniformly distributed in different angles. **(c)** Representative images showing the comparison between the direction of migration and the direction of cell polarity in CSCs and NSCCs. Scale bar: 20 *μm*. **(d)** The polarization directions of CSCs and NSCCs are consistent with the cell migration directions.
(TIF)

S4 Fig If membrane tension is not released, the body cannot exhibit new polarity in the simulation of the severing experiment. (TIF)

S5 Fig Schematic diagram of the free energy landscape of cell polarity. Two potential wells correspond to the polarized and nonpolarized states of the cell. The higher effective free energy barrier between the two states is overcome by cells with greater membrane tension only if the stimulation is sufficiently strong.
(TIF)

S6 Fig Sensitivity analysis of all parameters in the cell polarity model with phase field formulation. As the parameter values varied by 30%, the change in the maximum Rac-GTP concentration is less than 5% for all parameters, with the exception of the most sensitive parameter *K*_*1*_ (~25%), suggesting that our model is insensitive to the parameter values. The relatively high sensitivity of *K*_*1*_ is reasonable, as it represents the microscopic dissociation constant of the self-activation of Rac-GTP, which is the most significant production term for Rac-GTP (note the maximum production rate *c*_*1*_>*c*_*2*_, Equations 5, 6 and 7 and S2 Table).
(TIF)

S7 Fig Influence of the initial conditions on the polarized state. **(a-b)** Steady-state spatial profiles of Rac-GTP in the polarized state when the initial homogeneous concentrations of Rac-GTP and Rac-GDP are increased **(a)** or decreased **(b)** by 30% (compare with Fig 1b). The distribution of Rac-GTP increases (decreases) when the initial values increase (decrease). **(c)** Variations of the maximum and total Rac-GTP concentrations observed when the initial concentrations of Rac-GTP and Rac-GDP change by different percentages.
(TIF)

S1 Movie Cell polarity rotates as the direction of the stimulation gradient varies. (MOV)

S2 Movie When facing two competitive stimuli, the front under the stronger stimulus will finally ‘absorb’ the other front. (MOV)

S1 Table Values of the parameters in the cell polarity model with phase field formulation. (PDF)

S2 Table Values of the parameters in the traditional cell polarity model coupled with membrane tension. (PDF)
