# Supplementary material for: Exploring the inhibitory effect of membrane tension on cell polarization
Source: PLoS Comput Biol. 2017 Jan 30;13(1):e1005354. doi: 10.1371/journal.pcbi.1005354 (PMC5305267; doi:10.1371/journal.pcbi.1005354)
Supplement: S1 Text — (PDF) [file pcbi.1005354.s001.pdf]

Supporting Information

S1 Text. Traditional cell polarity model coupled with membrane tension

We also developed a traditional 2D cell polarity model coupled with membrane tension to verify the robustness of the mechano-chemical mechanism. In this model, we assume that a 2D cell represents the projection of a 3D cell on one plane; hence, the cell membrane overlaps with the cell cytosol, and both membrane-bound Rac-GTP and cytosolic Rac-GDP are distributed throughout the cell. The cell has the same shape as the phase field formulation, with a 10 μm diameter.

We added a term qfv to account for the positive feedback from F-actin (f) to Rac-GTP (v) (Fig 1a, [1-4]) to the original equations in the WP model (Equations S1a and b) and assume that the activation rate is linearly related to f. We add a third equation (Equation S1c) to incorporate the regulatory effects of Rac-GTP and membrane tension on F-actin polymerization. For the positive activation from Rac-GTP, we assume that the polymerization rate is linearly dependent on the concentration of Rac-GTP and the free polymerization rate of the branched F-actin is g. For the inhibitory effect of membrane tension, we apply the Brownian ratchet model and assume that the polymerization rate is proportional to the factor $\exp\left(-\frac{F\delta}{k_B T}\right)$ [5]. Here, *F* is primarily the force from membrane tension loaded on the filament [6, 7], $F\delta$ is the work done by adding one monomer against the load (δ is the half size of G-actin) [8], k_B is the Boltzmann constant and T is the temperature (310 *K*). We assume that $F = P/N$, where P denotes the pressure generated by membrane tension and N represents the filament density of F-actin which pushes the membrane, to link F with membrane tension and f [9]. We assume a linear relationship between N and the length density of F-actin with a converter parameter L =0.2 *μm*, which is regarded as the average persistent length of F-actin [7, 10], to calculate N. Therefore, $N = (f + f_0)/L$ (f_0 denotes the basal value of F-actin). We assume that P is linearly correlated with membrane tension (mt) and $P = \alpha mt$ ($\alpha = 1(\mu m^{-1})$). As defined in the main text, $mt(f) = mt_0\left(1 + \lambda\int_{\Omega_0} f\,ds\right)$, thus

$P = \alpha m t_0 \left(1 + \lambda \int_{\Omega_0} f ds\right) = P_0 \left(1 + \lambda \int_{\Omega_0} f ds\right)$, where $\lambda = \frac{1}{\int_{\Omega_0} f_0 ds}$. Thus, the exponential term is $\exp\left(-\frac{P\delta}{k_B T (f + f_0)/L}\right)$. We use an equivalent substitute f_e ($f_e = P\delta L/(k_B T) = \left(1 + \lambda \int_{\Omega_0} f ds\right) P_0 \delta L/(k_B T)$) for simplification, which is treated as an effective F-actin concentration. Finally, the rate of F-actin depolymerization (d) is assumed to be constant.

Based on the assumptions above, the dynamics of the traditional cell polarity model with membrane tension are described using the following equations:

$$\frac{\partial u}{\partial t} = D_u \nabla^2 u + \left(b + \frac{cu^2}{u^2 + K^2} + qf\right) v - ru \quad \text{(a)}$$

$$\frac{\partial v}{\partial t} = D_v \nabla^2 v - \left(b + \frac{cu^2}{u^2 + K^2} + qf\right) v + ru \quad \text{(b)} \qquad (1)$$

$$\frac{\partial f}{\partial t} = gu \exp\left(-\frac{f_e}{f + f_0}\right) - df \quad \text{(c)}$$

We apply a two-dimensional geometry with a no flux boundary condition to our model. In the simulation, we utilize a matrix (φ) to define the shape of the cell. The value of φ is 1 inside of the cell and 0 outside the cell. Moreover, the concentrations of Rac-GTP, Rac-GDP and F-actin are defined as $u\varphi$, $v\varphi$ and $f\varphi$, respectively. The diffusion of Rac-GTP and Rac-GDP is calculated when φ =1. The external stimuli are described as same as the stimuli (Equations 4-5) used in the cell polarity model with the phase field formulation. The initial values of Rac-GTP, Rac-GDP and F-actin for polarization are uniform. The values of all parameters are listed in S2 Table.

We ran the same type of simulations used with the cell polarity model with phase field formulation to test this traditional cell polarity model coupled with membrane tension. We confirmed that this model also generates similar features as the other previous published polarity models [11, 12], e.g., the formation of the steady-state spatial profiles of Rac-GTP (S1c Fig) and F-actin (S1d Fig) in a polarized cell in response to a transient gradient stimulus.

Furthermore, consistent with the cell polarity model with phase field formulation, this model generates results showing the inhibitory effect of membrane tension on cell polarity, e.g., the replication of Houk's aspiration-release experiment and severing experiment (Fig 4b and d).

Reference

1. Wang, F., *The Signaling Mechanisms Underlying Cell Polarity and Chemotaxis.* Cold Spring Harbor Perspectives in Biology, 2009. **1**(4): p. a002980-a002980.
2. Goehring, N.W. and S.W. Grill, *Cell polarity: mechanochemical patterning.* Trends in Cell Biology, 2013. **23**(2): p. 72-80.
3. Dawes, A.T. and L. Edelstein-Keshet, *Phosphoinositides and Rho Proteins Spatially Regulate Actin Polymerization to Initiate and Maintain Directed Movement in a One-Dimensional Model of a Motile Cell.* Biophysical Journal. **92**(3): p. 744-768.
4. Sasaki, A.T., et al., *Localized Ras signaling at the leading edge regulates PI3K, cell polarity, and directional cell movement.* The Journal of cell biology, 2004. **167**(3): p. 505-518.
5. Peskin, C.S., G.M. Odell, and G.F. Oster, *Cellular motions and thermal fluctuations: the Brownian ratchet.* Biophysical Journal, 1993. **65**(1): p. 316-324.
6. Xiong, Y., et al., *Mechanisms Controlling Cell Size and Shape during Isotropic Cell Spreading.* Biophysical Journal, 2010. **98**(10): p. 2136-2146.
7. Mogilner, A. and B. Rubinstein, *The Physics of Filopodial Protrusion.* Biophysical Journal, 2005. **89**(2): p. 782-795.
8. Mogilner, A. and G. Oster, *Force Generation by Actin Polymerization II: The Elastic Ratchet and Tethered Filaments.* Biophysical Journal. **84**(3): p. 1591-1605.
9. Mogilner, A. and L. Edelstein-Keshet, *Regulation of actin dynamics in rapidly moving cells: A quantitative analysis.* Biophysical Journal, 2002. **83**(3): p. 1237-1258.
10. Mogilner, A. and G. Oster, *Cell motility driven by actin polymerization.* Biophysical Journal, 1996. **71**(6): p. 3030-3045.
11. Jilkine, A. and L. Edelstein-Keshet, *A Comparison of Mathematical Models for Polarization of Single Eukaryotic Cells in Response to Guided Cues.* PLoS Computational Biology, 2011. **7**(4): p. e1001121.
12. Mori, Y., A. Jilkine, and L. Edelstein-Keshet, *Wave-Pinning and Cell Polarity from a Bistable Reaction-Diffusion System.* Biophysical Journal, 2008. **94**(9): p. 3684-3697.
