# Supplementary figures and images for: Exploring the inhibitory effect of membrane tension on cell polarization
Source: PLoS Comput Biol. 2017 Jan 30;13(1):e1005354. doi: 10.1371/journal.pcbi.1005354 (PMC5305267; doi:10.1371/journal.pcbi.1005354)

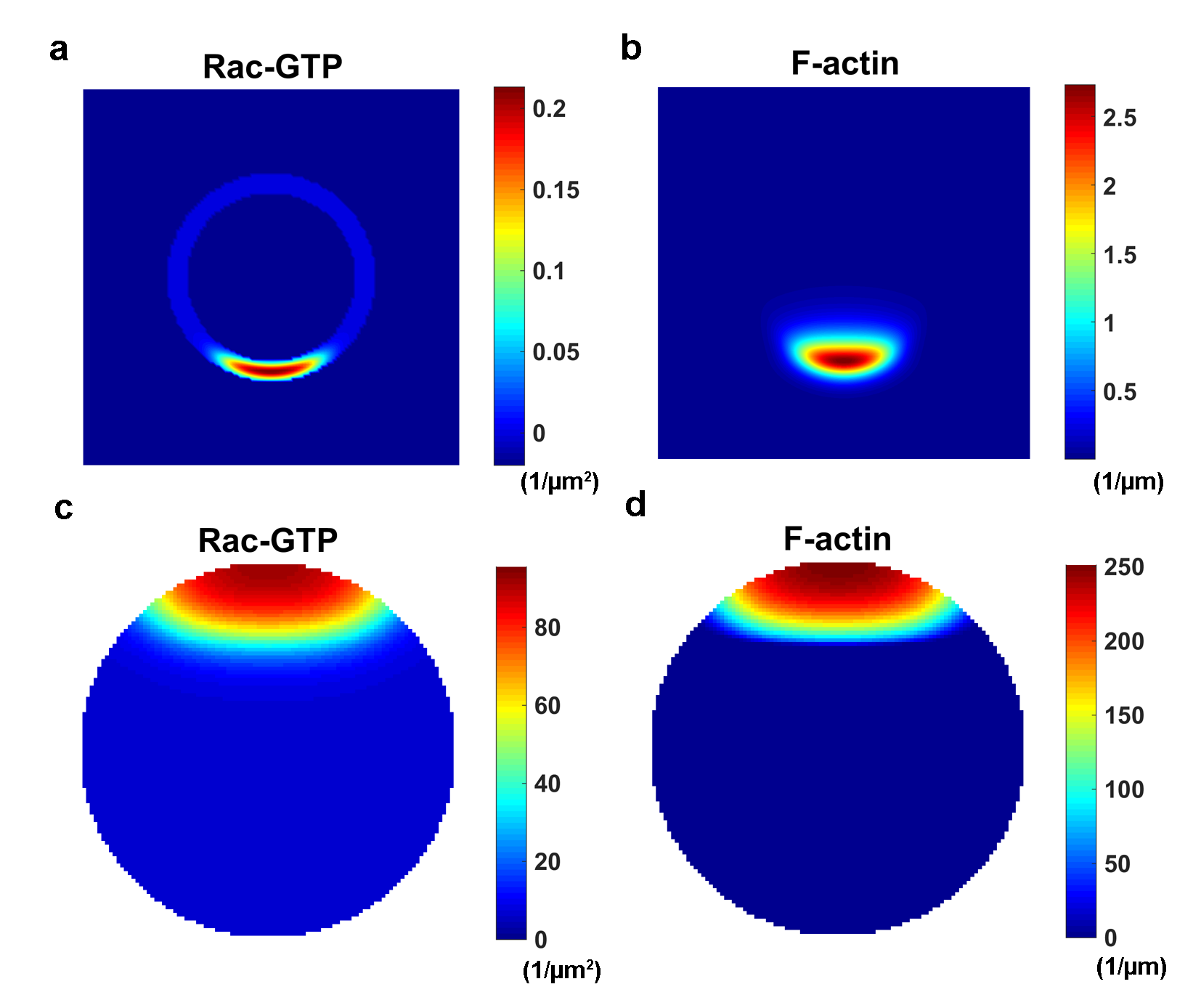

Supplement: S1 Fig — (a-b) Steady-state spatial distribution of Rac-GTP (a) and F-actin (b) during the spontaneous polarization of a cell in response to noise simulated using the cell polarity model with phase field formulation. (c-d) Steady-state spatial profiles of Rac-GTP (c) and F-actin (d) in a polarized cell in response to a transient gradient stimulus calculated using the traditional polarity model. (TIF) [file pcbi.1005354.s002.tif]

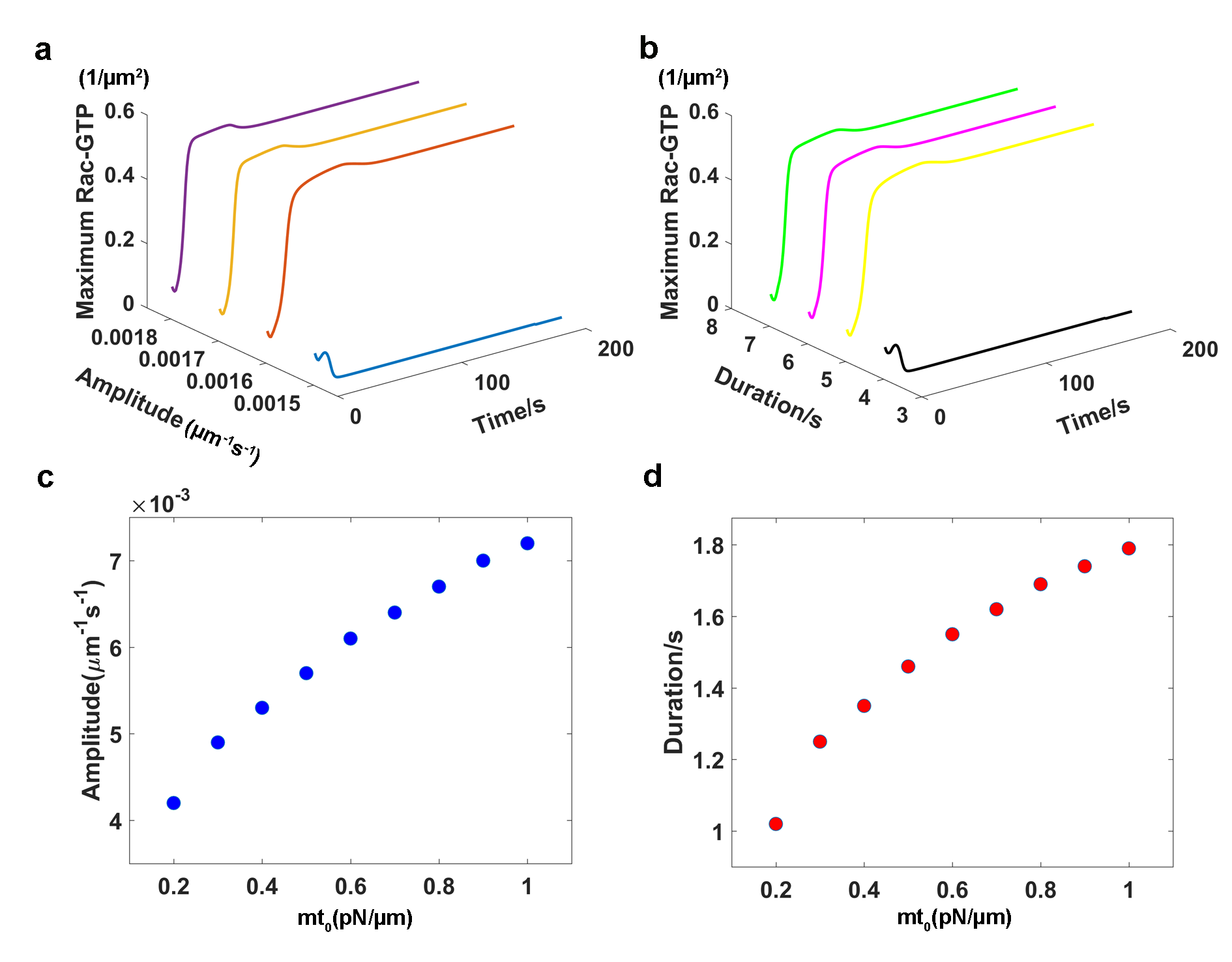

Supplement: S2 Fig — (a) The dynamics of the maximum Rac-GTP concentration at a fixed duration and varying amplitudes of the stimuli. When the amplitude of the stimulus is below a threshold, the cell cannot polarize (blue line). The other lines show the polarization dynamics as the amplitudes exceed the threshold. (b) The dynamics of the maximum Rac-GTP concentration in the stimulation with varied durations and a fixed amplitude. When the duration of the stimulus is below a threshold, the cell cannot polarize (black line). The other lines show the polarization dynamics as the durations exceed the threshold. (c) For duration-fixed stimuli, the threshold of the stimulation amplitude required for polarization increases as membrane tension increases. (d) For amplitude-fixed stimuli, the threshold of the stimulation duration increases as membrane tension increases. (TIF) [file pcbi.1005354.s003.tif]

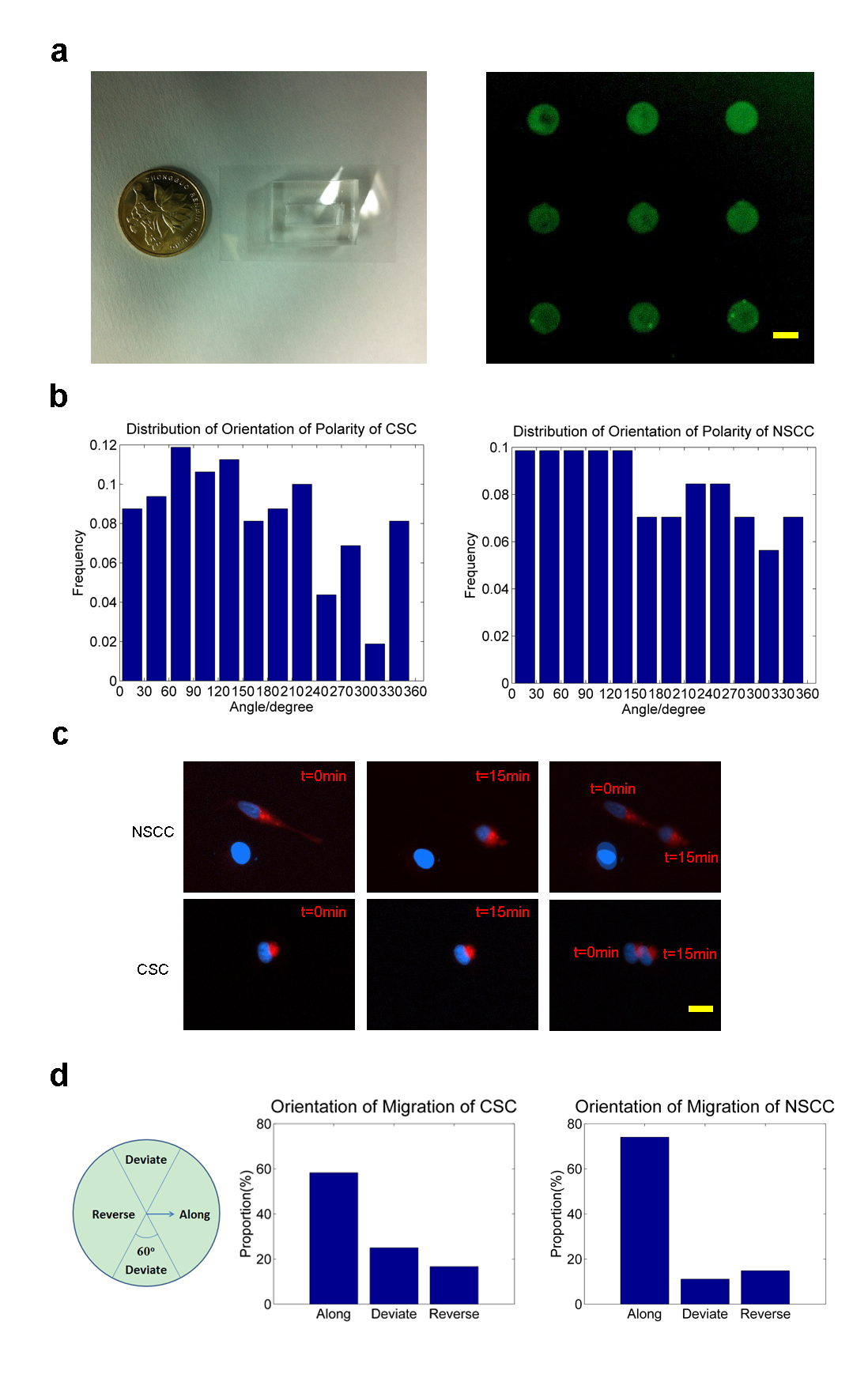

Supplement: S3 Fig — (a) Pictures of the microstructure chip and images of the fluorescent, coated ECM patterns. Scale bar: 20 μm. (b) The orientations of polarized CSCs and NSCCs are uniformly distributed in different angles. (c) Representative images showing the comparison between the direction of migration and the direction of cell polarity in CSCs and NSCCs. Scale bar: 20 μm. (d) The polarization directions of CSCs and NSCCs are consistent with the cell migration directions. (TIF) [file pcbi.1005354.s004.tif]

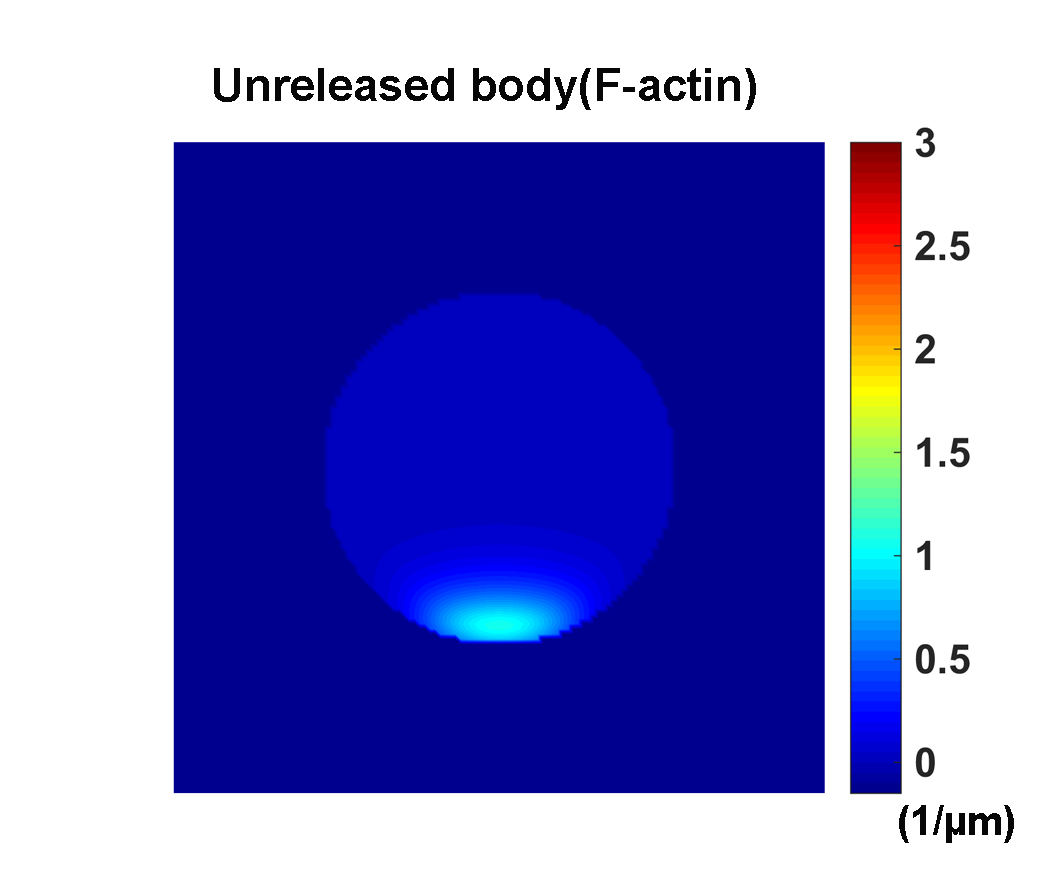

Supplement: S4 Fig — (TIF) [file pcbi.1005354.s005.tif]

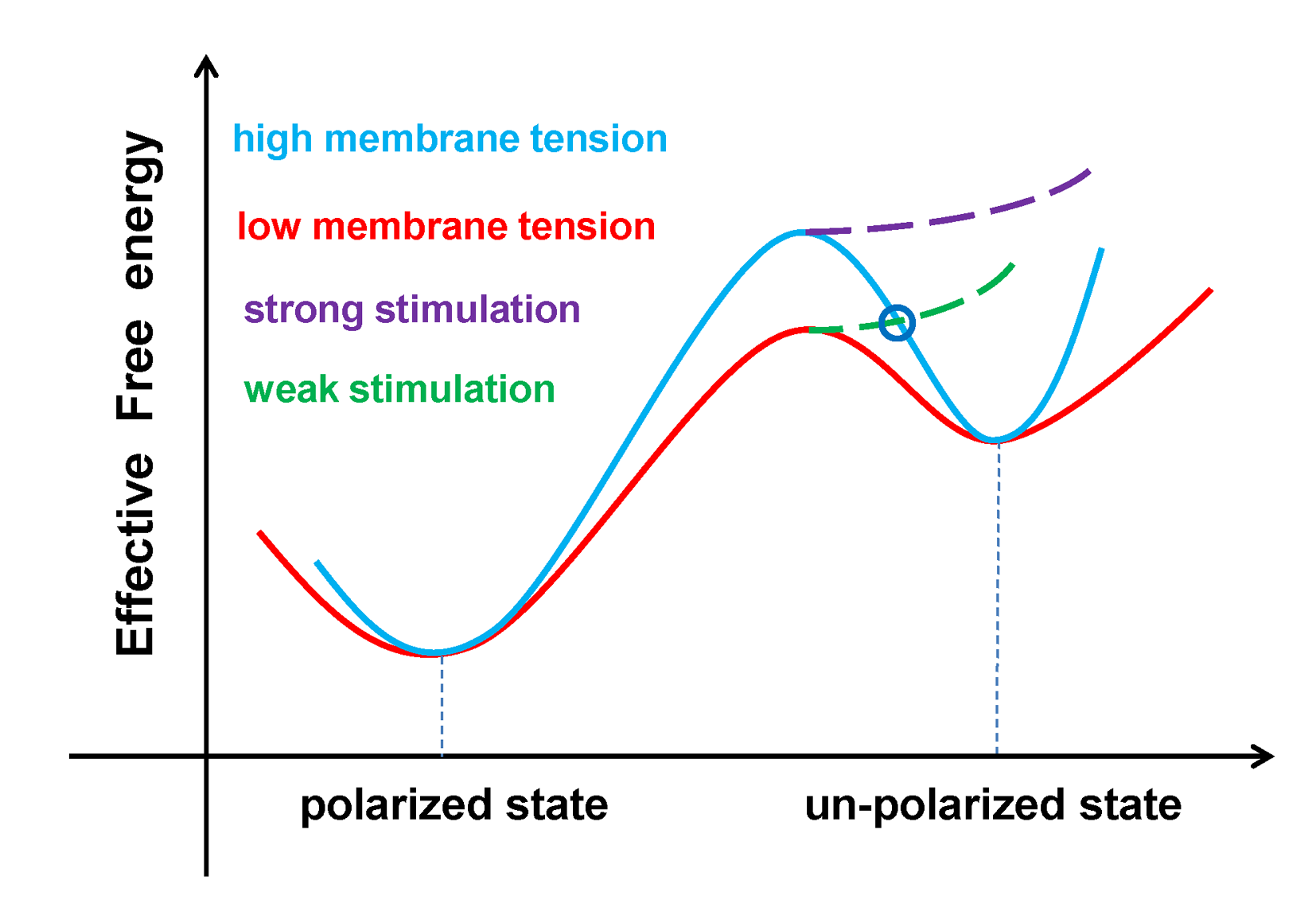

Supplement: S5 Fig — Two potential wells correspond to the polarized and nonpolarized states of the cell. The higher effective free energy barrier between the two states is overcome by cells with greater membrane tension only if the stimulation is sufficiently strong. (TIF) [file pcbi.1005354.s006.tif]

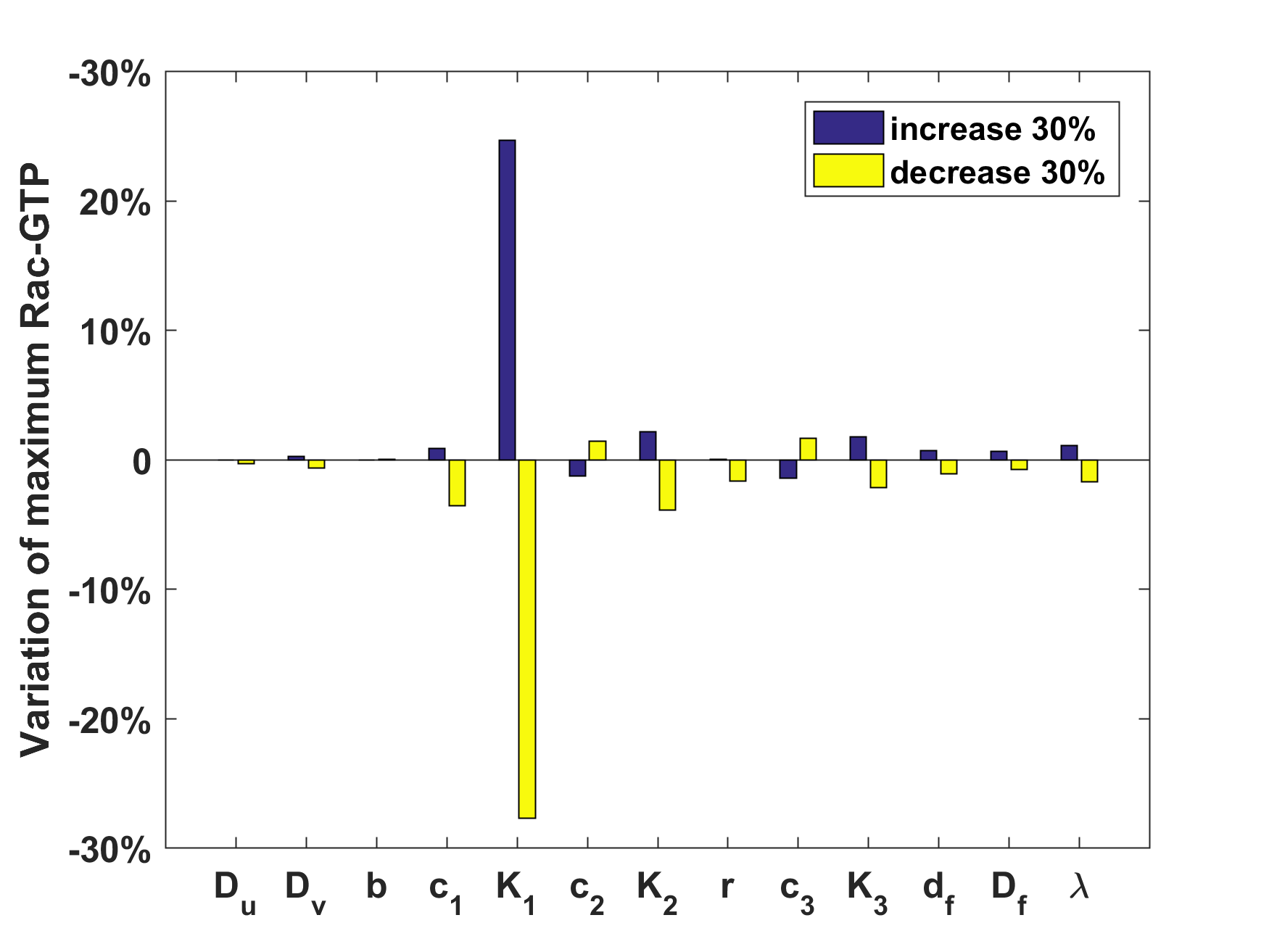

Supplement: S6 Fig — As the parameter values varied by 30%, the change in the maximum Rac-GTP concentration is less than 5% for all parameters, with the exception of the most sensitive parameter K1 (~25%), suggesting that our model is insensitive to the parameter values. The relatively high sensitivity of K1 is reasonable, as it represents the microscopic dissociation constant of the self-activation of Rac-GTP, which is the most significant production term for Rac-GTP (note the maximum production rate c1>c2, Equations 5, 6 and 7 and S2 Table). (TIF) [file pcbi.1005354.s007.tif]

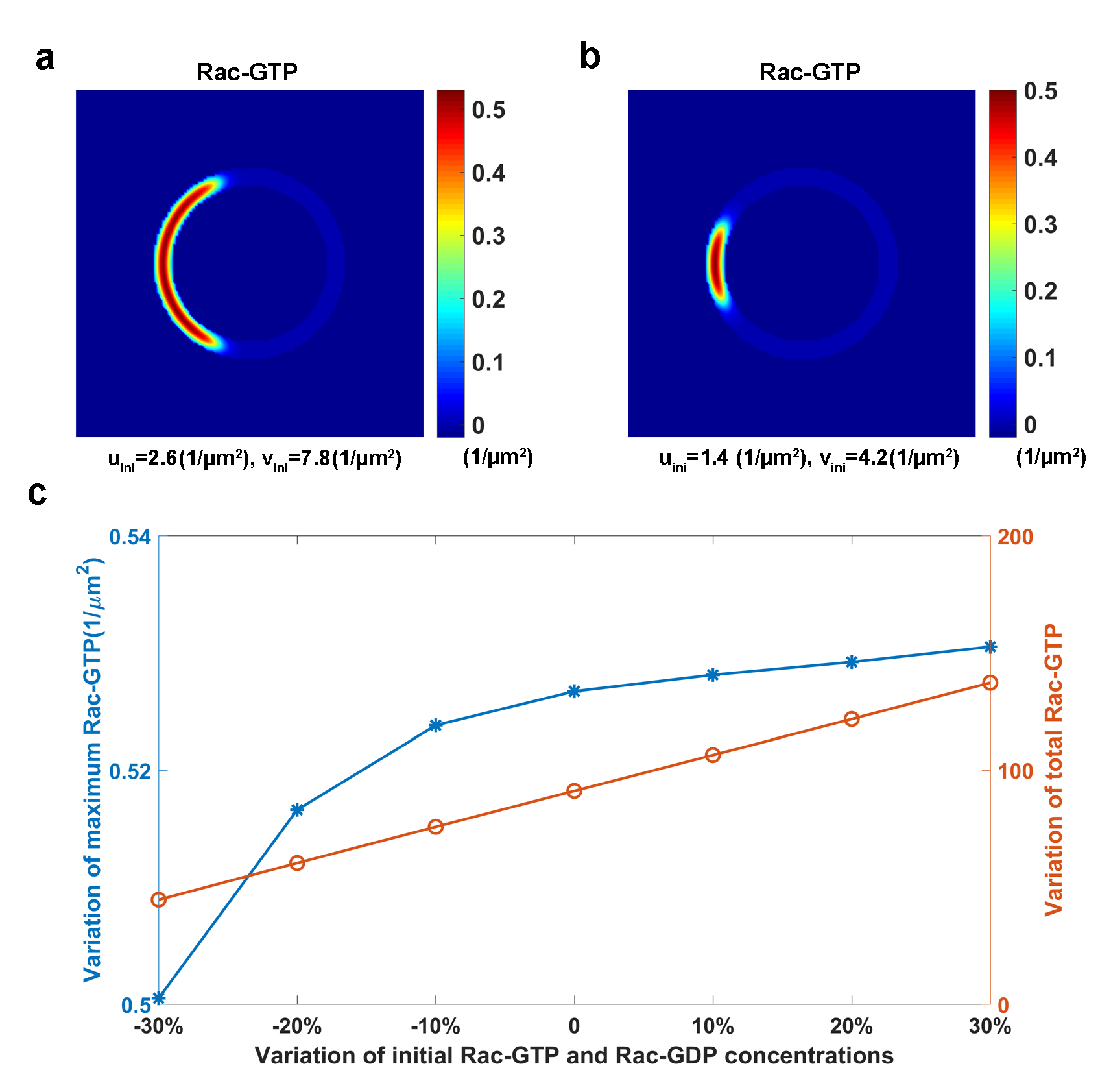

Supplement: S7 Fig — (a-b) Steady-state spatial profiles of Rac-GTP in the polarized state when the initial homogeneous concentrations of Rac-GTP and Rac-GDP are increased (a) or decreased (b) by 30% (compare with Fig 1b). The distribution of Rac-GTP increases (decreases) when the initial values increase (decrease). (c) Variations of the maximum and total Rac-GTP concentrations observed when the initial concentrations of Rac-GTP and Rac-GDP change by different percentages. (TIF) [file pcbi.1005354.s008.tif]
