# Supplementary material for: Exploring the inhibitory effect of membrane tension on cell polarization
Source: PLoS Comput Biol. 2017 Jan 30;13(1):e1005354. doi: 10.1371/journal.pcbi.1005354 (PMC5305267; doi:10.1371/journal.pcbi.1005354)
Supplement: S1 Table — (PDF) [file pcbi.1005354.s011.pdf]

S1 Table. Values of Parameters in the cell polarity model with phase field formulation.

Parameter	Figure 1 (b)-(f), Figure 2 (a)-(c), Figure 3 (a)-(c).	Figure 4 (a)	Figure 4 (b)	Unit	*Reference*
D_u	0.1	0.1	0.1	$\mu m^2/s$	*[1]*
D_v	50	50	50	$\mu m^2/s$	*[1]*
b	0.001	0.001	0.001	s^{-1}	--
c_1	3	0.5	2.4	s^{-1}	*[2]*
K_1	6	10	12	μm^{-2}	--
c_2	0.2	0.48	0.5	s^{-1}	--
K_2	3	3	3	μm^{-2}	--
r	1.4	1	1.4	s^{-1}	*[3]*
c_3	6	6	6	$\mu m^{-}2/s$	--
K_3	3	3	3	μm^{-2}	--
d_f	1	1	1	s^{-1}	--
D_f	0.8	0.8	0.8	$\mu m^2/s$	*[4]*
λ	0.95	0.95	0.95	μm^{-1}	
mt_0	0.2-1	0.2-1	0.5	*pN/μm*	--
K_F	1	1	1	*pN/μm*	--

"--" means not available in references

Reference

1. Postma, M., et al., *Chemotaxis: signalling modules join hands at front and tail.* EMBO reports, 2004. **5**(1): p. 35-40.
2. Mori, Y., A. Jilkine, and L. Edelstein-Keshet, *Wave-Pinning and Cell Polarity from a Bistable Reaction-Diffusion System.* Biophysical Journal, 2008. **94**(9): p. 3684-3697.
3. Dawes, A.T. and L. Edelstein-Keshet, *Phosphoinositides and Rho Proteins Spatially Regulate Actin Polymerization to Initiate and Maintain Directed Movement in a One-Dimensional Model of a Motile Cell.* Biophysical Journal, 2007. **92**(3): p. 744-768.
4. Shao, D., H. Levine, and W.-J. Rappel, *Coupling actin flow, adhesion, and morphology in a computational cell motility model.* Proceedings of the National Academy of Sciences, 2012. **109**(18): p. 6851-6856.
