# Supplementary material for: Exploring the inhibitory effect of membrane tension on cell polarization
Source: PLoS Comput Biol. 2017 Jan 30;13(1):e1005354. doi: 10.1371/journal.pcbi.1005354 (PMC5305267; doi:10.1371/journal.pcbi.1005354)
Supplement: S2 Table — (PDF) [file pcbi.1005354.s012.pdf]

S2 Table. Values of Parameters in the traditional cell polarity model coupled with membrane tension.

Parameter	*Value*	*Unit*	*Reference*
D_u	*0.1*	*μm²/s*	*[1]*
D_v	*10*	*μm²/s*	*[1]*
b	*0.05*	s^{-1}	*[2]*
c	*1*	s^{-1}	*[2]*
q	*0.0012*	*μm/s*	--
K	*60*	μm^{-2}	--
r	*1*	s^{-1}	*[3]*
g	*0.1*	*μm/s*	*[4]*
f_e	*10~70*	$\mu m/\mu m^2$	--
f_0	*10-30*	$\mu m/\mu m^2$	--
d	*0.03*	s^{-1}	*[3]*
P_0	*100*	$pN/\mu m^2$	*[5]*
δ	*2.7*	*nm*	*[6]*
L	*1*	*μm*	*[7]*

"--" means not available in references

Reference

1. Postma, M., et al., *Chemotaxis: signalling modules join hands at front and tail.* EMBO reports, 2004. **5**(1): p. 35-40.
2. Mori, Y., A. Jilkine, and L. Edelstein-Keshet, *Wave-Pinning and Cell Polarity from a Bistable Reaction-Diffusion System.* Biophysical Journal, 2008. **94**(9): p. 3684-3697.
3. Dawes, A.T. and L. Edelstein-Keshet, *Phosphoinositides and Rho Proteins Spatially Regulate Actin Polymerization to Initiate and Maintain Directed Movement in a One-Dimensional Model of a Motile Cell.* Biophysical Journal, 2007. **92**(3): p. 744-768.
4. Abraham, V.C., et al., *The actin-based nanomachine at the leading edge of migrating cells.* Biophysical Journal, 1999. **77**(3): p. 1721-1732.
5. Xiong, Y., et al., *Mechanisms Controlling Cell Size and Shape during Isotropic Cell Spreading.* Biophysical Journal, 2010. **98**(10): p. 2136-2146.
6. Peskin, C.S., G.M. Odell, and G.F. Oster, *Cellular motions and thermal fluctuations: the Brownian ratchet.* Biophysical Journal, 1993. **65**(1): p. 316-324.
7. Mogilner, A. and B. Rubinstein, *The Physics of Filopodial Protrusion.* Biophysical Journal, 2005. **89**(2): p. 782-795.
